# Children with idiopathic toe walking display differences in lower limb joint ranges and strength compared to peers: a case control study

**DOI:** 10.1186/s13047-022-00576-x

**Published:** 2022-09-12

**Authors:** Antoni Caserta, Prue Morgan, Marnee J. McKay, Jennifer N. Baldwin, Joshua Burns, Cylie Williams

**Affiliations:** 1 Monash Health Community, 140-155 Sladen Street, Cranbourne, VIC Australia; 2 Department of Physiotherapy, Monash University, Frankston, Victoria Australia; 3 University of Sydney School of Health Sciences, Faculty of Medicine and Health, Sydney, New South Wales Australia; 4 School of Health Sciences, College of Health, Medicine and Wellbeing, and Priority Research Centre for Physical Activity and Nutrition, The University of Newcastle, Callaghan, New South Wales Australia; 5 University of Sydney School of Health Sciences, Faculty of Medicine and Health & Children’s Hospital at Westmead, Sydney, Australia; 6 Peninsula Health, Allied Health, Frankston, Victoria Australia; 7 School of Primary and Allied Health, Monash University, Frankston, Victoria Australia

**Keywords:** Idiopathic toe walking, Strength, Range of motion, Hand-held dynamometry, Rehabilitation, Gait

## Abstract

**Background:**

Idiopathic toe walking (ITW) is an exclusionary diagnosis. There has been limited exploration of lower limb active range of motion and strength measures in children with ITW. This researched aimed to determine any differences in lower limb muscle active range of motion and strength in children who have ITW, compared to normative data collected from children who displayed typical gait.

**Methods:**

Children were recruited with had a diagnosis of ITW, aged between 4 and 10 years, and no recent treatment. Data collected included parent reported data such as time spent toe walking, percentage of time spent toe walking, and clinician collected data such as age, height and weight. Joint ranges of motion and strength measures were collected by an experience clinician. Active and weight bearing joint ranges of motion were evaluated with a goniometer or digital inclinometer. Lower limb muscle strength measures were evaluated with a hand-held dynamometer. Published normative data sets were used for comparison. Measures were analysed with regression analyses to determine differences between groups in different measures, considering measures known to impact range and strength. Odds ratios (OR), 95% confidence intervals (CI) and *p* values were reported.

**Results:**

Twenty-six children with ITW participated. Reduced weight bearing ankle range of motion, when measured with the knee bent, was associated with being in the ITW group (*p* = 0.009), being older (*p* < 0.001) and weighing less (p < 0.001). Reduced ankle plantar flexion range was only associated with being in the ITW group (*p* = 0.015). For all lower limb strength measures, excluding hip external rotation, children who displayed greater strength, did not toe walk (*p* < 0.002), were older (*p* < 0.001) and weighed more (*p* < 0.014). with ITW.

**Conclusion:**

Children with ITW displayed reduced overall plantar and dorsiflexion at the ankle, compared to non-toe walking children. Reduced plantarflexion is children with ITW has not been described before, however reduced dorsiflexion is commonly reported. Children with ITW were weaker in many lower limb measures, even when age and weight were considered. This should lead clinicians and researchers to pay greater attention to lower limb strength measures in this population.

## Background

Toe walking is commonly described when a person walks with a limited or lack of heel strike at the initial contact phase of the gait cycle. This can be a common variation during gait acquisition [1]. Consistent heel strike typically appears during gait and is usually present in most children by the age of 5 years [2]. There are many conditions known to cause toe walking gait including trauma or injury to the lower limb, neurological conditions or developmental or behavioural conditions [3, 4]. Specific conditions that account for the majority of toe walking gait presentations include cerebral palsy, muscular dystrophy, autism spectrum disorders, global developmental delay, lower limb injury or tumours [4].

Idiopathic toe walking (ITW) is diagnosed when all other suspected diagnoses are eliminated [4]. This diagnosis has an estimated prevalence in 5% of healthy children [5] and is diagnosed in both sexes [6]. ITW is most commonly associated with ankle equinus [7]. In other populations who exhibit equinus part of a diagnosis or disability, equinus is thought to contribute to lower limb or foot pain [8], poor performance in sport, and low participation in exercise [9]. There is no known studies linking the impact of equinus to these concerns in the ITW population.

There are few treatments for ITW with robust evidence supporting their use, however allied health professionals encourage treatment that may be manual therapy, such as stretching, or motor control interventions, such as home exercise programs [10]. There are also no toe walking specific patient reported outcome measures or consensus measures known at present to guide treatment choice. Whilst muscle strengthening is a key feature of many recommended therapies, at present, observational and interventional studies investigating the efficacy of therapeutic interventions consistently include gait analysis and lower limb range of motion as primary outcome measures. The use of tools to measure strength associated with ITW have only been described within three studies [6, 11, 12], with no consistency in describing or measuring strength or only examining one muscle group (crossing the ankle). More commonly, studies consistently report a change in ankle range of motion as a key indicator of intervention study success [4].

Muscle strength measurement is an important outcome measure assessed in clinical practice. In other conditions known to result in toe walking, foot and ankle strength and equinus are commonly linked, such as seen in children with Charcot Mariet Tooth Disease [13]. In children who have Cerebral Palsy, those who display muscle weakness also find it difficult to complete simple or complex movement patterns such as walking, running, hopping, skipping, climbing and jumping- all play patterns that are common and important in childhood [14]. While these conditions have a neurological and genetic component to their impact, they are also a population that toe walks and often are used for comparisons.

There has been limited exploration of joint ranges of motion in the lower limb in children who have ITW, with previous studies primarily focusing on ankle range. There has been no measurement of lower limb muscle group strength, other than the ankle dorsiflexors or hip flexors in children with ITW [4]. The primary aim of this study was to identify if there is a difference in the ankle joint range of motion between those with ITW and neurotypical peers. The secondary aims was to investigate any differences between other joints in the lower limb and if there were any association of ITW with lower limb strength measures.

## Methods

### Study design and participants

This study was a case control design and approved by Monash Health Human Research Ethics Committee (HREC number: 15405A). Ethics approval for The 1000 Norms Project was provided by National Health and Medical Research Council of Australia Centre for Research Excellence in Neuromuscular Disorders (NHMRC 1031893). All parents/carers of participants provided written informed consent, and child participants assented.

### Study population

Participants were recruited from private practice clinics, public health outpatient and community clinics. Participants were eligible if they were between the aged 4–16 years, visually demonstrated a toe walking gait, and were diagnosed with idiopathic toe walking gait by a multidisciplinary clinic with medical and allied health team. If potential participants were toe walking and had not seen the multidisciplinary clinic, they were screened with a validated exclusionary tool [3]. The screener was clinician researcher who had > 8 years working in public health community-based paediatric gait screening clinics.

Participants were excluded if they had lower limb pain at the time of initial screening, had previous ITW treatment with ankle foot or full length orthotics, recent prescription of stretches or strengthening program targeted at the lower limb that the child was adhering to at a dosage that was deemed potentially having clinical impact, or serial casting or Botulinum Toxin-A as part of their ITW treatment within the past 12 months.

Normative raw data was sourced from two data sets. Strength and active range of motion data was collected from the 1000 Norms Project. The 1000 Norms Project is an observational study investigating outcome measures of self-reported health and physical function in 1000 healthy individuals aged 3 to 101 years [15]. A secondary normative data source was used for comparing the weight bearing lunge test in a straight leg position between children who have ITW with their age matched peers [7].

### Outcome measures

The following demographic data was collected from the parent: child’s age (years)**,** sex, weight (kg), height (m), parent-reported duration of toe walking (years), parental estimate of percentage of time awake that the child toe walks (% of walking).

The primary outcome of interest was the weight bearing ankle joint range of motion. Additional lower limb range measures, and strength measures were also assessed (Table 1).

**Table 1:** Range of motion and strength measures

Region of interest	Assessment Position	Limb position and assessment task or movement
Range of motion		
Hip external rotation	Seated, hip and knee flexed to 90°.	Turn the leg towards the middle,
Hip internal rotation	Seated, hip and knee flexed to 90°.	Turn the leg out to the side,
Hip flexion	Supine, knees extended, hips in neutral	Bring the knee towards the chest
Knee extension	Supine, legs extended, hips in neutral	Straighten the knee
Knee flexion	Supine, legs extended, hips in neutral	Bend the knee so the foot moves towards the buttock
Ankle plantarflexion	Seated, knees and hips at 90°	Bend the ankle, pointing the toes to the ground
Ankle dorsiflexion (Weight bearing)	Standing in lunge position facing wall (leg straight)	Keep foot and heel flat on the floor, foot straight and lean towards keeping leg straight centred knee over the midline of the foot
Ankle dorsiflexion (Weight bearing)	Standing in lunge position facing wall (knee straight)	Keep foot and heel flat on the floor, foot straight and bend knee towards wall, centred over the midline of the foot
Strength		
Hip external rotators	Upright sitting	Hips and knees flexed at 90° and externally rotate
Hip internal rotators	Upright sitting	Hips and knees flexed at 90° and internally rotate
Hip abductors	Supine lying	Legs extended, hip in approximately 10°abduction and abduct
Knee flexors	Upright sitting	Knee in 60° flexion and flex knee
Knee extensors	Upright sitting	Knee in 60° flexion and extend knee
Ankle dorsiflexors	Long sitting	Ankle in mid-range plantarflexion and dorsiflex
Ankle plantarflexors	Long sitting	Ankle in plantarflexion and plantarflex

Weight bearing ankle joint range of motion was assessed with a calibrated digital inclinometer. Active joint ranges of motion were assessed with a universal goniometer. The starting positions, limb positions and assessment task/movement were performed as per the 1000 Norms Project protocol, a summary of this is described in Table 1.

Lower limb muscle strength testing was undertaken with the Citec handheld dynamometer (Citec dynamometer CT 3001, CIT technics, Groningen, the Netherlands). Each participant was assessed using the “make” technique to measure strength and were directed to exert a maximal force against the hand-held dynamometer [16]. The starting position, and limb position and movement assessed are described in Table 1. The universal goniometer, and digital inclinometer (Laser Depot, Adelaide, Australia) and hand-held dynamotor have all demonstrated high reliability when used according to the set measurement protocol [16].

### Study procedure

The principal researcher (AC) had experience in utilising these measurement techniques (AC), however prior to data collection, had peer support to match technique with measurement protocols with an experienced physiotherapist and podiatrist. The principal researcher was responsible for all participant testing. For the range of motion measures, each participant was asked to perform the movement to their end range and hold while the tester recorded the active or weightbearing range of motion. For the strength measures, each participant had a practice trial at submaximal effort, then were instructed to perform three maximal voluntary contractions lasting three to 5 seconds each. Given the age of participants, rapport with the participant was obtained prior to testing. Instructions and encouragement were individualised to the personality and patients’ age to account for difference cognitive abilities. Participants were given a resting period of 10 seconds in between each contraction. All data were entered into an online spreadsheet. It was pre-planned that where a participant was unable to perform the measure, no data were recorded for that item and treated as missing data.

### Data analysis

Data were analysed with the use of Stata 13 (Stata Corp, College Station Texas). Descriptive synthesis of demographic data were completed. Anthropometric measures were described in means (Standard Deviations = SD) or frequencies (%) after confirming normal distribution of data. As ITW is only diagnosed when the child toe walks symmetrically, only right leg measures were used. This has been found to satisfy assumptions of data independence where there is likelihood of high correlation between two limbs [15].

We originally explored any differences between groups using logistic regression. The data from the ITW group and normative group were originally compared using univariate logistic regression analysis to determine any group differences in each individual measure. Backwards step multivariable linear regression analysis was then conducted for each individual range of motion or strength variable taking into account other variables identified as impacting range of motion or strength. Where there were variables that were highly intercorrelated (*r* > 0.7), for example, height and weight, only one variable was included to avoid multicollinearity. The preliminary multivariable model for each measure were built with variables identified in univariate analysis that revealed a value of *p* ≤ 0.2. During the analysis, variables were then removed based on least significant fit in a backward stepwise fashion. This backward step removal continued until all remaining model variables had a *p*-value < 0.05 [17]. Regression coefficients (Coef) were reported to understand direction of the relationship, and 95% confidence intervals (CI) were also reported.

A sample size of 26 participants was calculated to achieve 80% power, and to detect an effect size of 0.81 as a result of differences in ankle range of motion (primary outcome) between the ITW cohort and their non-toe walking peers using an α criterion of 0.05 [7].

## Results

Sixty-seven potential participants were screened for inclusion after expressing an interest in participating in the study. Participants were excluded due to having a potential condition known to cause toe walking (*n* = 17), receiving an interventional treatment within 12 months of the study (*n* = 5), declined to participate or data collected was not meaningful due to the participant not wanting to perform tasks even after parent consent and child assented (*n* = 16), not observed to toe walk at screening appointment (*n* = 3). Twenty-six children diagnosed with ITW entered the study and completed full range of motion and strength data collection. Three parents were unable to provide an accurate age of onset of independent walking or toe walking. Participants in the ITW group started independent walking at an average age of 14.0 months (*n* = 23, SD = 4.3, range = 8 to 24 months). Parents described onset of toe walking at an average age of 16.52 months (*n* = 23, SD = 5.5 SD, range 8 to 25 months,). At the time of assessment, the average length of time participants had been toe walking was 56.4 months (*n* = 23, SD = 20.4, range 24 to 112 months). Twenty-four of the 26 participants (92%) were right-handed.

Comparison data were sourced for the same age group using the 1000 Norms Project dataset [16], and from weight bearing leg lunge test data for the leg straight normative data set [7]. Records from a total of 136 participants were thus obtained for children aged between 4 and 10 years. Characteristics of the groups, number (%), mean (SD), median (Interquartile range (IQR), of demographics, ranges of motion and strength measures of both participants with ITW and normative groups are provided in Table 2.

**Table 2:** Characteristics of the groups, number (%), mean (SD), median (IQR), of demographics, ranges of motion and strength measures (Normative data versus ITW data)

Characteristics	Normative Group Mean (SD) or *n(%)*, *Range*	Idiopathic toe walking Group Mean (SD) or *n(%)*, *Range*	Coef [95% CI] p
Age (years)	6.9 (2.0), *4 to 10*	6.3 (1.8)*, 4 to 10*	−0.18 [− 0.40, 0.05] 0.127
Sex (male)	*68 (50%)*	*17 (65%)*	−0.64 [−1.2, 0.24] 0.154
Height (m)	1.3 (0.1), *0.9 to 1.6*	1.2 (0.1), *1.0 to 1.5*	−4.19 [−7.59, − 0.80] 0.016
Weight (kg)	27.6 (8.8), *14.1 to 54.2*	24.0 (6.6), *15.0 to 40.0*	−0.06 [− 0.12, 0.01] 0.056
**Lower limb range of motion(**^**o**^**)**			
Hip internal	41.3 (8.6), 20 to 67	37.4 (14.4), 15 to 70	−0.04 [− 0.09, 0.01] 0.064
Hip external	35.2 (11.8), 9 to 65	31.6 (8.5), 16 to 65.5	0.04 [−0.01, 0.08] 0.074
Hip flexion	132.4 (9.7), 106 to 160.5	131.5 (16.2), 80 to 157	− 0.01 [− 0.05, 0.03] 0.708
Knee extension	3.4 (3.4), −5.5 to 14.5	2.9 (9.2), −18 to 25	− 0.02[− 0.11, 0.07] 0.620
Knee flexion	144.8 (5.6), 129 to 156.5	146.0 (6.8), 134 to 162	0.03 [−0.04, 0.11] 0.354
Ankle plantarflexion	62.6 (8.1), 33.5 to 85.0	57.9 (12.3), 35 to 80	−0.05 [− 0.10, − 0.01] 0.018
Ankle dorsiflexion (straight leg)	32.8 (4.4), 26.1 to 43.2^a^	26.3 (10.5), 2.3 to 45.9	−0.12 [− 0.21, − 0.03] 0.009
Ankle dorsiflexion (bent knee)	32.9 (7.1), 16 to 49	29.1 (10.7), 5.5 to 50.5	− 0.06 [− 0.12, − 0.01] 0.029
**Lower limb strength (N)**			
Hip internal rotation	73.0 (29.7), 20 to 168	*49.6 (21.8), 11 to 93*	−0.04 [− 0.06, − 0.02] < 0.001
Hip external rotation	54.1 (20.4), 12 to 111	44.6 (19.6), 7 to 84	− 0.03 [− 0.05, − 0.01] 0.027
Hip abduction	40.1 (17.3), 8 to 77	61.5 (24.1), 20 to 127	− 0.05 [− 0.07, − 0.02] < 0.001
Knee extension	127.1 (43.2), 45 to 265	81.9 (29.2), 27 to 133	− 0.04 [0.05, − 0.02] < 0.001
Knee flexion	95.2 (32.4), 25 to 212	70.1 (32.4), 14 to 176	− 0.03 [− 0.05, − 0.01] 0.001
Ankle dorsiflexion	96.9 (34.5), 24 to 203	61.1 (34.7), 11 to 158	− 0.04 [− 0.06, 0.02] < 0.001
Ankle plantarflexion	164.7 (49.7), 66 to 285	72.3 (35.5), 12 to 177	−0.06 [0.08, − 0.04] < 0.001

^a^Normative data from 30 children (age range 4 to 8 years)

There were limited differences between the range of motion measures of children in the ITW compared to the normative data. Only the ankle plantarflexion range of motion measured in a non-weight bearing position (Coef = − 0.05, 95% CI = − 0.10 to − 0.01,*p* = 0.018) and dorsiflexion in a weight bearing with both the leg straight (Coef = − 0.12, 95%CI = − 0.21 to − 0.03, *p* = 0.009), and the knee bent (Coef = − 0.06, 95%CI = − 0.12 to − 0.01, *p* = 0.029) were significantly different between children with ITW and normative peer data, with those with ITW demonstrating less range of motion in these selected outcomes. There were significant differences found between the measures of children in the ITW compared to the normative peer data for all lower limb strength measures (*p* < 0.03), with children with ITW having less muscle strength. Table 2 displays the results of each muscle group strength measure differences between the groups. Table 3 outlines the range of motion and strength variables that were associated with having an ITW gait pattern, and where variables such as age and weight were an influence.

**Table 3:** Multivariable analysis of strength and range of motion variables associated with toe walking status, age and weight

	Group^ Coef, [95% CI] p	Age (Years) ^α^ Coef, [95% CI] p	Weight^α^ Coef, [95% CI] p
**Range of motion measure** (^o^)			
Ankle plantarflexion	−4.66, (−8.40, −0.91), 0.015		
Ankle dorsiflexion (bent knee)	−4.32, (− 7.55, − 1.09) 0.009	1.90, [0.91, 2.90] < 0.001	− 0.44, [− 0.67, − 0.21] < 0.001
**Strength measures (N)**			
Hip internal rotation	− 14.74, [− 22.71, − 6.76] < 0.001	5.37, [2.90, 7.85], < 0.001	1.47, [0.90, 2.03] < 0.001
Hip abduction	−14.38, [− 20.74, − 8.02] < 0.001	4.92, [2.9, 6.89], < 0.001	1.09, [0.64, 1.54] < 0.001
Knee extension	−33.18, [45.75, − 20.61] < 0.001	4.88, [0.98, 8.77], < 0.001	2.48, [1.59, 3.37] 0.014
Knee flexion	−16.39, [− 26.68, − 6.10] 0.002	5.87, [2.68, 9.06], < 0.001	1.37, [0.64, 2.10] < 0.001
Ankle dorsiflexion	−25.88, [− 36.11, − 15.64] < 0.001	6.96, [3.79, 10.14], < 0.001	1.52, [0.79, 2.24] < 0.001
Ankle plantarflexion	− 78.77, [− 92.92, − 64.62] < 0.001	9.73, [5.34, 14.12], < 0.001	2.07, [1.07, 3.07] < 0.001

^ Normative versus ITW, ^α^ Increase in number

Weight bearing ankle range of motion, when measured with the knee bent, was associated with ITW and the child’s weight and age. This meant that children who had greater range in this position did not toe walk (Coef = − 4.32, 95% CI = -7.55 to − 1.09, *p* = 0.009), were older (Coef = 1.90, 95%CI = 0.91 to 2.90, *p* < 0.001) and weighed less (Coef = 0.44, 95%CI = -0.67 to − 0.21, *p* < 0.001). Ankle plantar flexion range was only impacted by toe walking, with children measuring greater plantar flexion range if they did not toe walk (Coef = − 4.66, 95%CI = -8.40 to − 0.91, *p* = 0.015). Less hip internal rotation, hip abduction, hip flexion, knee flexion, knee extension, ankle plantar flexion and ankle dorsiflexion strength were all associated with ITW. This meant for all lower limb strength measures, excluding hip external rotation, children who displayed greater strength, did not toe walk (*p* < 0.002), were older (*p* < 0.001) and weighed more (*p* < 0.014). with ITW.

## Discussion

This study provides new information about lower lower limb strength and range of motion measures in children with an ITW gait compared to typically developing children. A finding that children with ITW were not as strong in their lower limbs as their peers, may either be the reason that they first initiate the gait pattern, or a key result arising from their gait condition. Finding minimal differences between lower limb joint ranges of motion, other than the ankle range of motion in those with ITW versus normative peers, is also a novel finding. This highlights that children with ITW may not develop proximal joint tightness above the ankle from their altered gait pattern.

Finding differences in ankle range of motion was in concordance with other studies investigating ITW populations [6, 7, 18]. A novel finding in this study was not only less ankle dorsiflexion in the ITW group, but also less plantarflexion range, resulting in a decrease in total ankle range of motion. Our study also identified that all hip and knee active ranges of motion were not influenced by ITW when height, weight and age were considered. Ankle joint range of motion reduction has previously been described in relationship to total upper and lower limb joint ranges of motion in an ITW cohort [6]. Children with ITW were up to 3.2 times more likely to display reduced ankle joint dorsiflexion range of motion, than children who walked with a heel toe gait [6]. This cohort of children with ITW, also did not display reduced ranges of motion when upper and lower limb ranges were combined [6].

Few studies have used the weight bearing lunge test to investigate differences in ankle range, despite its increased preference for use in neurological populations where ongoing contracture monitoring is required [13]. Using this measure with the leg in a straight position revealed a difference in dorsiflexion ankle range in a prior study with children who had ITW and their typically developing peers [7]. However, any difference between the groups significantly decreased when age and weight were considered. It is possible that increasing age, with a corresponding increase in weight impacts joint range of motion, and may be why less toe walking is observed as children get older [5, 19].

Muscle strength is a key ingredient in a complex system enabling children to complete functional movement patterns such as walking, running, jumping, hopping, skipping or climbing. As children mature, we should expect muscle strength in particular leg muscles to increase with maturation and progressive task acquisition. This is not always the case however for children with many medical conditions or disabilities. Muscle weakness can be a sign of or predispose a number of pathologies [13, 16]. Previous strength-related tasks have been explored with small cohorts of children with ITW. Children with ITW have demonstrated challenges with complex movements that required greater strength, particularly evident in younger children [20]. Other ITW observational or interventional studies either had different methodology, did not have a neurotypical, non-toe walking control group, or analysis techniques [6, 11, 12]. These studies collated limited strength measures and only collected data from the targeted muscle group being treated.

There were a number of limitations to our study. Other publications that include measures of strength commonly collect the data in Newton meters, and as a measure of torque. This may be more relevant and associated with strength at different heights [21], or when measures are taken longitudinally. The decision to use newtons as a measure output in our study, was to enable data matching muscle strength as described within the 1000 Norms protocol [22]. This resulted in raw data in Newtons as preplanned as our comparable data. We also did not consider use of passive joint range of motion. The challenge of variability in measures, rater reliability and lack of participant level comparative data of passive measures meant we did not consider its use for this study, We that due to this, clinicians may have different clinical observations in practice. We also acknowledge that any mean differences between the children with ITW and normative population data used may be small. It is unknown if these differences are clinically significant when collected in isolation to other functional or quality of life measures.

The vast majority of reasons for toe walking gait are neurological in origin [23]. These is building evidence of ITW also resulting from subtle neurological differences between toe walking and non-toe walking peers [24, 25]. Children with mild spastic diplegia and children with ITW have demonstrated similar kinematics and electromyography during gait analysis [26]. Therefore, it may be warranted to consider future research comparing ranges of motion and strength among difference cohorts of children who have toe walking gait, regardless of its cause. These findings may lead to better understanding of the toe walking gait establishment, it’s progression and may be vital to improve treatments depending on presentation rather treatment mapped to diagnosis.

Overall, finding differences in lower limb strength between children with ITW and children that do not toe walk is promising for future interventional research. However, this study may not be powered appropriately to identify strength differences, as the sample size was developed based on the primary outcome of ankle range of motion. No other studies have reported strength differences in specific lower limb joints other than ankle dorsiflexion, therefore findings from this present study may inform calculations of a meaningful sample size for relevant future research. Finding that children with ITW were weaker in many lower limb measures, even when age and weight are considered, should lead clinicians and researchers to pay greater attention to strength measurement and monitoring in this population.

Although this present study compares and contrasts the lower limb impairments with a large normative dataset, we have been unable to compare these results with any previous studies on the ITW population. This highlights opportunities for future research to consider developing a suite of tests that should be considered with studies including children with ITW. This research supports a suite of strength and range of motion measures from the whole lower limb, but these may be collected functionally together with measures of impact on quality of life or participation. Having a standard suite of clinically appropriate measures will enable future treatment trials to collect similar measures thus allowing future systematic reviews to compare results that matter to families.

## Conclusion

This study revealed detailed lower limb ranges of motion and strength characteristics about children with ITW. Participants exhibited widespread lower limb weakness and less total ankle range of motion than their typically developing peers. This finding may encourage researchers to develop a more comprehensive minimum data set to use in ITW studies, and clinicians to consider more detailed strength assessment and strength training as part of any intervention.

## Data Availability

Request for further details of the data set and queries relating to data sharing arrangements may be submitted to Antoni Caserta (Antoni.caserta@monashhealth.org). Aggregate or summarised data may be shared based on reasonable request.

## Abbreviations

ITW: Idiopathic toe walking
OR: Odds ratios
CI: Confidences interval
Kg: Kilograms
M: Metres
