# Correction: Children with idiopathic toe walking display differences in lower limb joint ranges and strength compared to peers: a case control study

**DOI:** 10.1186/s13047-022-00585-w

**Published:** 2022-11-04

**Authors:** Antoni Caserta, Prue Morgan, Marnee J. McKay, Jennifer N. Baldwin, Joshua Burns, Cylie Williams

**Affiliations:** 1 Monash Health Community, 140-155 Sladen Street, Cranbourne, VIC Australia; 2 Department of Physiotherapy, Monash University, Frankston, Victoria Australia; 3 University of Sydney School of Health Sciences, Faculty of Medicine and Health, Sydney, New South Wales Australia; 4 School of Health Sciences, College of Health, Medicine and Wellbeing, and Priority Research Centre for Physical Activity and Nutrition, The University of Newcastle, Callaghan, New South Wales Australia; 5 University of Sydney School of Health Sciences, Faculty of Medicine and Health & Children’s Hospital at Westmead, Sydney, Australia; 6 Peninsula Health, Allied Health, Frankston, Victoria Australia; 7 School of Primary and Allied Health, Monash University, Frankston, Victoria Australia

**Correction: J Foot Ankle Res 15, 70 (2022)**

**https://doi.org/10.1186/s13047-022-00576-x**

Following publication of the original article [1], author found two minor errors in Table 2, specifically:

- Hip abduction - Normative Group Mean SD should be 61.5.
- Hip abduction - Idiopathic toe walking Group Mean SD should be 40.1.

The correction does not have any effect on the results or conclusions of the paper. The original article has been corrected.
